# An *In Silico* Study of the Differential Effect of Oxidation on Two Biologically Relevant G-Quadruplexes: Possible Implications in Oncogene Expression

**DOI:** 10.1371/journal.pone.0043735

**Published:** 2012-08-22

**Authors:** William J. D. Stebbeds, Joseph Lunec, Lee D. Larcombe

**Affiliations:** Cranfield Health, Cranfield University, Cranfield, Bedfordshire, United Kingdom; Peking University Health Science Center, China

## Abstract

G-quadruplex structures, formed from guanine rich sequences, have previously been shown to be involved in various physiological processes including cancer-related gene expression. Furthermore, G-quadruplexes have been found in several oncogene promoter regions, and have been shown to play a role in the regulation of gene expression. The mutagenic properties of oxidative stress on DNA have been widely studied, as has the association with carcinogenesis. Guanine is the most susceptible nucleotide to oxidation, and as such, G-rich sequences that form G-quadruplexes can be viewed as potential “hot-spots” for DNA oxidation. We propose that oxidation may destabilise the G-quadruplex structure, leading to its unfolding into the duplex structure, affecting gene expression. This would imply a possible mechanism by which oxidation may impact on oncogene expression. This work investigates the effect of oxidation on two biologically relevant G-quadruplex structures through 500 ns molecular dynamics simulations on those found in the promoter regions of the c-Kit and c-Myc oncogenes. The results show oxidation having a detrimental effect on stability of the structure, substantially destabilising the c-Kit quadruplex, and with a more attenuated effect on the c-Myc quadruplex. Results are suggestive of a novel route for oxidation-mediated oncogenesis and may have wider implications for genome stability.

## Introduction

G-quadruplexes (G4-DNA) are a class of secondary structures formed from guanine rich sequences that have been implicated in telomere maintenance, genome stability and gene (including oncogene) expression [1], [2], [3]. In recent years both bioinformatics analyses and *in vitro* methods have shown G4-DNA abundance in upstream promoter regions and at telomeric ends [4], [5], and the potential effects of the folding and unfolding of these structures *in vivo* [3], [6], [7], [8]. Within upstream promoter regions, G-quadruplexes typically take on the form of intramolecular structures consisting of three tetrads of four guanines, stacked on top of each other; each tetrad is stabilised by eight Hoogsteen hydrogen bonds between the Guanines and the π- π (stacking) interactions between the tetrads (reviewed in [9]). Two of the most well studied G4-DNA structures are those found in the promoter regions of the proto-oncogenes c-Myc and c-Kit.

Elevated expression levels of c-Myc are associated with self-sufficiency of cancer cells, through regulation of cellular proliferation, differentiation and apoptosis [10], [11]. The G-quadruplex structure found in the nuclease hypersensitive element (NHE) III region [12] upstream of the gene has been widely linked to regulation of c-Myc expression.

The NHE III is a C rich, 27 base pair long sequence and is located approximately 100 bp upstream of the P1 promoter of c-Myc [13] and has been shown to repress transcription of c-Myc when in a non-canonical state [14]. This was then shown to be due to G-quadruplex formation by the use of a G4-DNA binding ligand, TMPyP4, which stabilised the G-quadruplex in the NHE III region and decreased c-Myc expression [15].

Similarly, the c-Kit proto-oncogene is also associated with the self-sufficiency of cancer cells, and has been found to be increased in a variety of cancers [3] it is now a target in the treatment of gastrointestinal tumours. Two G-quadruplex forming motifs (GQMs) have been discovered in the promoter region. Structures have been elucidated for both c-Myc and c-Kit-related G4-DNA motifs which are available in the Protein Data Bank [12], [16], [17].

The mutagenic properties of oxidative stress on DNA have been widely studied, as has their association with carcinogenesis. Of particular interest is the association of oxidation with the up- and down-regulation of transcription when mutations occur within the promoter region of cancer associated genes (reviewed in [18]. The oxidation of deoxyguanosine to 8-oxo-2′deoxyguanosine (8-oxodG) is the most frequent effect when DNA is under oxidative stress [19] and indeed, 8-oxo-dG is used as a biomarker for DNA oxidative damage [20]. As such, the G-rich sequences that form G-quadruplexes can be viewed as potential “hot-spots” for DNA oxidation.

The effect of oxidation on the thermodynamic stability of G4-DNA has not been widely studied. However studies performed on all parallel tetramolecular structures *in vitro* [21], [22] have shown that oxidation is well tolerated and, in certain circumstances, can promote the formation and stability of the G-quadruplex structure.

However, recent studies [23] investigating the effect of oxidation on the stability of telomeric G-quadruplex DNA have found that the incorporation of 8-oxo-dG destabilises the quadruplex structure, with potential effects on telomere maintenance. Additional *in vitro* studies of G-quadruplexes have revealed stability is dependent on factors such as loop length [24], [25], sequence [25] and number of tetrads [26], and so it is likely that the varied stability observed for different G4-DNA motifs may affect the extent to which these structures are susceptible to chemical modification, such as oxidation.

We propose, that oxidation may also decrease the stability of intramolecular G-quadruplexes found in upstream promoter regions; suggesting a possible mechanism by which oxidation may impact on oncogene expression.

Here we present an *in silico* molecular dynamics study of the effects of 8-oxodG presence in the tetrad structures of the c-Kit and c-Myc-related G4-DNA motifs. Through the longest published simulations performed on the c-Kit and c-Myc G4-DNA structures to date, we observe disruption of the G-quadruplex structure on incorporation of just a single 8-oxodG and propose therefore, oxidation as a mechanism for altering gene expression for genes with GQM-containing promoters – of particular relevance to oncogene expression.

## Results and Discussion

**Figure 1:**
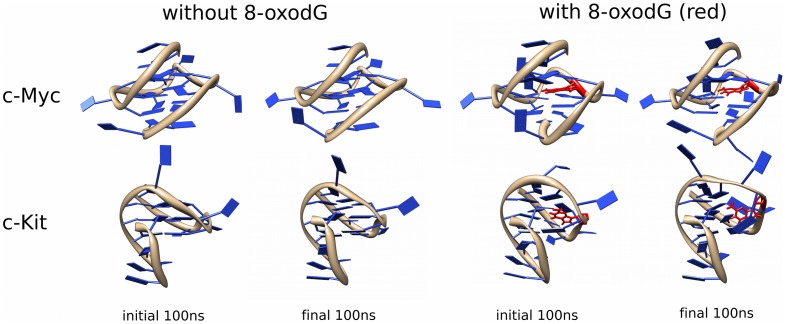
Comparison of the effect of 8-oxodG on the c-Myc and c-Kit G-quadruplexes. Most prevalent clusters (NMRclust) observed in the initial 100 ns and final 100 ns of the MD simulations are represented for c-Myc and c-Kit structures with and without the inclusion of 8-oxodG. Top row shows c-Myc first simulated without 8-oxodG (initial and final 100 ns) followed by c-Myc simulated with 8-oxodG at position G9 (shown in red) (initial and final 100 ns). Similarly, the second row shows c-Kit first simulated without 8-oxodG (initial and final 100 ns) followed by c-Kit simulated with 8-oxodG at position G7 (shown in red) (initial and final 100 ns).

**Figure 2:**
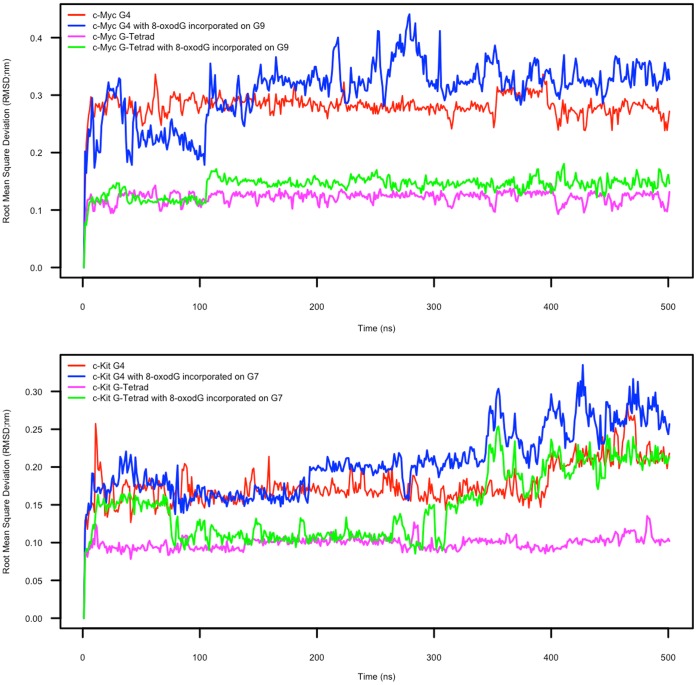
RMSD plots of the G-quadruplex structures with and without 8-oxodG incorporated. Plots of all-atom (red and blue) and tetrad-specific (magenta and green) RMSD for the c-Myc (top graph) and c-Kit (bottom graph) G-quadruplex structures with and without 8-oxodG incorporated. Data was extracted from the simulation trajectories using the g_rms tool, part of the GROMACS package and plotted using R.

Molecular Dynamics simulations were carried out on equilibrated c-Myc and c-Kit promoter G4-DNA structures using parameters approximating physiological conditions for 500 ns. Representations of the first and last 100 ns of the MD simulations are presented in Figure 1, showing snapshots representing the largest groups of similar structures (clusters) observed during those times. To simulate the effect of oxidation, both G4-DNA structures had a single 8-oxo-dG nucleotide incorporated into the central tetrad (G9 in c-Myc and G7 in c-Kit; shown in red in figure 1). The 500 ns simulations of the non-oxidised c-Myc and c-Kit (Figure 1, top left and bottom left structures, respectively) G-quadruplexes exhibit good stability throughout the simulations. All-atom root mean square deviation (RMSD) calculations for the structures, and specific calculations focussed on the atoms of Guanines involved in tetrad formation (tetrad-specific RMSD) (Figure 2) give low values for both the non-oxidised c-Myc and c-Kit G4-DNA structures (average all-atom RMSD of 0.282 nm with average tetrad-specific RMSD of 0.112 nm; and average all-atom RMSD of 1.78 Å with average tetrad specific RMSD of 0.105 nm respectively). Hydrogen bonding calculations (calculated for atoms from the Guanine bases involved in tetrad formation with a maximum distance of 0.35 Å and 60° cut-off) support these findings, as both non-oxidised structures presented an average number of Hydrogen bonds of 24, throughout the 500 ns simulation, accounting for all the Hoogsteen hydrogen bonds expected (8 per tetrad, for 3 tetrads - data not shown). These results point to the remarkable stability of these structures, particularly the Hoogsteen-bonded tetrads, at near physiological conditions.

The extent of the effects of incorporating 8-oxodG, as observed in Figures 1 and 2, appears to be dependent on the structure of the G-quadruplex. In the c-Myc G4-DNA structure the clusters observed in Figure 1 (top right structures) show that the G-tetrads retain π- π stacking with adjacent tetrad(s) and that each guanine, although fluctuating, does not appear to deviate in relation to the remaining guanines in the G-tetrad. In contrast, the 8-oxodG appears to twist in relation to the tetrad into which it was incorporated, however it still does not appear to affect the surrounding bases. The loop regions however, appear to fluctuate substantially with the closest loops to the 8-oxodG twisting outward, increasing the distance from the affected tetrad.

This observation is confirmed by the RMSD data (Figure 2) in which tetrad-specific RMSD is relatively low (averages of 1.19 Å and 1.47 Å for the final 100 ns for the normal and 8-oxodG-containing structures, respectively), although there is a more pronounced difference between the all-atom RMSD values of the normal and 8-oxodG-containing c-Myc quadruplexes (averages of 2.72 Å and 3.29 Å for the final 100 ns, respectively). The difference between the hydrogen bonding data further illustrates differences apparent from the macroscopic observations, as the 8-oxodG-containing c-Myc G-quadruplex retains only 22 hydrogen bonds throughout the simulation, indicating a loss of 2 Hydrogen bonds; one between the 8-oxodG base and each of the two adjacent Guanines.

Inclusion of 8-oxodG into the c-Kit-related G4-DNA structure (Figure 1, bottom right structures) had a less pronounced effect on the loop regions, with the only observable difference from the normal structure being that the nucleotides in the long loop (closest loop to the 8-oxodG inclusion site) show greater fluctuation, increasing the distance from the oxidised base. However, the Guanines involved in tetrad formation suffered substantial distortion over the course of the simulation. At around 300 ns the 8-oxodG within the central tetrad begins to twist in relation to the other Guanines, with this distortion spreading to the Guanines directly above and below in the stack and causing them to begin to move away from the remaining tetrads. This distortion becomes more apparent over the course of the simulation and appears to affect both the stacking of the tetrads and the Hoogsteen hydrogen bonding between the distorted Guanines and the tetrad.

These observations are supported by the RMSD plots (Figure 2) in which there is an increase in the tetrad-specific RMSD of the 8-oxodG-containing c-Kit structure compared to the normal, with average RMSD values of 0.210 nm and 0.142 nm respectively over the last 100 ns of the simulation. As with the c-Myc G4 structure, the c-Kit all-atom RMSD also shows increased values with respect to 8-oxodG, and to a greater extent than observed for the c-Myc G4-DNA (averages of 0.268 nm and 0.218 nm for the final 100 ns for the 8-oxodG and normal structures respectively). Hydrogen bonding estimation supports the RMSD and macroscopic observations made. During the final 100 ns of simulation, the c-Kit structure retains an average of only 16 Hoogsteen hydrogen bonds, consistent with the 8-oxodG and those Guanines directly above and below twisting away and partially loosing Hydrogen bonding with the rest of the stacked tetrads.

Principal component analysis (PCA) is a common multivariate analysis technique that can be used to identify large-scale collective motions of atoms and separate significant motion from background thermodynamic fluctuation [27]. This analysis provides eigenvectors (principal components) corresponding to directions of motion, and often a large proportion of the motility of the analysed molecule can be explained by a few eigenvectors with the highest eigenvalues.

**Figure 3:**
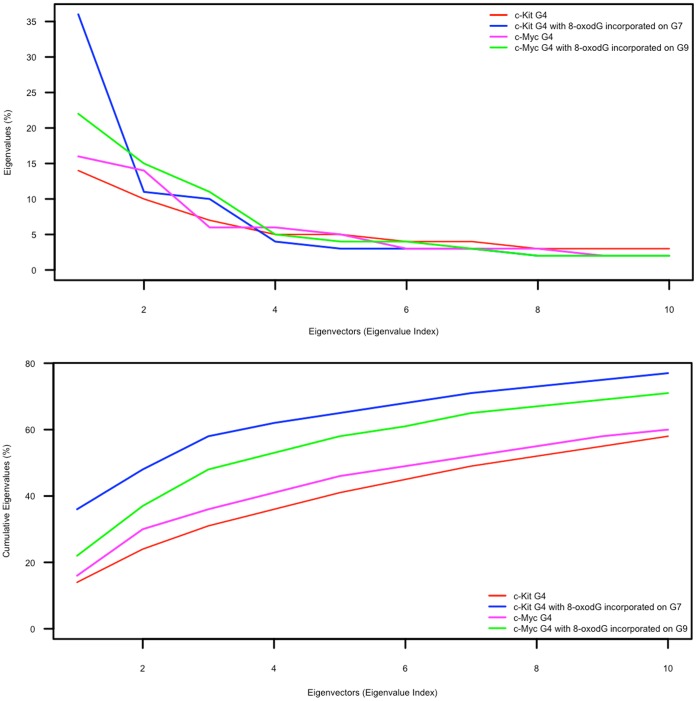
PCA of the G-quadruplex structures with and without 8-oxo-dG incorporated. Graph plotting percentage of motility explained by eigenvalues against eigenvectors (principal components) of the entire G-quadruplex structure (top) and cumulative percentage (bottom). Principal component analysis (PCA) was performed on the Molecular Dynamics trajectories using the g_covar and g_anaeig tools, part of the GROMACS package and plotted using R.

The percentage of motility as explained by eigenvectors with the 10 highest eigenvalues is presented in Figure 3; it plots the eigenvalues corresponding to the 10 eigenvectors as a percentage of eigenvalues for the total eigenvectors (Figure 3, top) and as a cumulative percentage (Figure 3, bottom). This analysis shows that the first 3 eigenvectors account for a large proportion of the motility of the 8-oxodG-containing c-Myc and c-Kit G4-DNA structures (approximately 50% and 60% respectively), but account for a smaller proportion of the normal c-Myc and c-Kit G4-DNA structures (approximately 35% and 30% respectively).

For both 8-oxodG-containing and normal G4-DNA, the structures corresponding to the extremes of the eigenvectors fit the macroscopic observations. The eigenvalues of eigenvectors for the normal G4-DNA structures are lower than those of the 8-oxodG-containing structures, and correspond to fluctuations of loop nucleotides and small distortions in the backbone of the loops. For the 8-oxodG-containing c-Myc quadruplex, the first 2 eigenvectors correspond to the distortion of both the loop nucleotides (rotating around the backbone) and the movement of the backbone of the loops away from the 8-oxodG. In the case of 8-oxodG-containing c-Kit structure the first eigenvector represents the distortion observed in the G-tetrads, with the 8-oxodG and the Guanines directly above and below it twisting and moving away from the remaining Guanines in the tetrads. The second eigenvector represents the distortions caused to the loop nucleotides, as observed macroscopically.

Considering macroscopic and PCA analyses together, it appears that although inclusion of 8-oxodG causes the c-Myc quadruplex to distort, the effects are principally seen in the loop regions; whereas in the case of inclusion of 8-oxodG in the c-Kit quadruplex, not only is distortion more apparent (both macroscopically and through data analysis), but the distortion is localised to the G-tetrads.

Several thermodynamic studies on the stability of G4-DNA have revealed the extent to which structural characteristics affect stability (reviewed in [9]), indicating that the two most import factors are the Hoogsteen hydrogen bonding between Guanines involved in tetrad formation and the π- π (stacking) interactions between adjacent tetrads [27]. Considering this it becomes apparent that the effect of 8-oxodG incorporation is more detrimental to the c-Kit quadruplex than the c-Myc structure, as the former appears to lose a quarter of the Hoogsteen hydrogen bonds as well as stacking of the tetrads. This suggests that the sensitivity of a G-quadruplex structure to oxidation is likely dependent on structural characteristics known to affect stability *in vitro*, such as loop length [25], [28] and sequence [26] and number of tetrads [27].

Reduced stability of G4-DNA is likely to have biological implications. The G-quadruplex selective RecQ helicases, WRN and BLM, have been observed to preferentially unwind quadruplex substrates containing 8-oxodG [29]; a G-quadruplex structure destabilised by oxidative stress is likely to be more susceptible to unwinding. Importantly, previous studies have shown that the expression of a number of oncogenes potentially mediated by G-quadruplex motifs is inhibited by stabilisation of G4-DNA, including the c-Kit [30] and c-Myc [15]oncogenes. This inhibition would suggest that destabilisation of G4-DNA through oxidative stress may be a possible mechanism for the overexpression or activation of such oncogenes, and offers a tentative explanation for previous studies showing apparent enrichment of G4-DNA motifs in genes affected by Hydrogen peroxide treatment [31], and where Benzoyl peroxide has been shown to specifically oxidise Guanine double and triple repeat sequences in dsDNA affecting regulation of tumour suppression genes and oncogene expression [32].

Significantly, considering the prevalence of GQMs in oncogene promoter regions [3], and that the transition of Guanine to 8-oxodG is a common occurrence when DNA is under oxidative stress *in vivo* [20], the effect on G4-DNA described here could be widespread. The results presented suggest that chemical modification of just a single Guanine involved in tetrad formation in intramolecular G-quadruplexes to 8-oxo-2′deoxyguanosine may be sufficient to destabilise the G4-DNA structure, suggesting a mechanism by which oxidative stress and exposure to reactive oxygen species may have a direct influence on oncogene expression, with wider implications for gene regulation and genomic stability.

This has been a short study and therefore has several limitations. Due to limited compute resource, we have not attempted to study the effect of oxidation at different positions within the G4-DNA structure, and neither have we evaluated the effect of multiple oxidation sites on the same g-quadruplex structure that could reveal a dose-dependent response from G-quadruplex DNA to oxidative stress. Additionally, as with any purely *in silico* study of course, the results should ideally be complemented by *in vitro* or *in vivo* experiments observing the effect of oxidative stress on possible G-quadruplex regulated genes in a biological context.

However, we feel that the current work describes an intriguing *in silico* observation that oxidation may affect the stability of G4-DNA structures, and that this could hint towards a novel route for the association between oxidative stress and carcinogenesis. It is our hope that these preliminary observations will be of interest to the wider community and stimulate debate and further research on this topic. To this end, we make the GROMACS port of the ParmBSC0 force field available to facilitate the wider use of molecular dynamics applied to DNA structural studies.

## Materials and Methods

All simulations were performed using GROMACS 4.5.3 [33]. Parameters for the simulations were based on the AMBER ParmBSC0 force field [34], ported to GROMACS by the authors – available on request. The PDB structure files for the c-Myc and c-Kit promoter G4-DNA structures were PDB ID: 1XAV and PDB ID: 2O3M, respectively. Parameters for the 8-oxodG nucleotide were taken from the Bryce Group at the University of Manchester (http://www.pharmacy. manchester.ac.uk/bryce/amber) and ported to GROMACS 4.5.3.

The methodology for both the molecule preparation and the simulations performed were adapted from that of Haider and Neidle (2010) [35] All structures were solvated in water, counterionised in KCl to a K+ concentration of 100 mM, and subsequently equilibrated, simulating physiological conditions at 310 K. Molecular dynamics (MD) simulations were then performed (isothermic, isobaric 500ns unrestrained simulation in explicit solvent and counterions) on the c-Kit and c-Myc G-quadruplex structures with and without 8-oxo-dG incorporated into the central tetrad.

Data was analysed using tools built in to the GROMACS package and are explained when used. Representative Clusters from trajectories were obtained using NMRclust [36] algorithm and images were captured using UCSF Chimera [37].
